# COVID-19 with acute cholecystitis: a case report

**DOI:** 10.1186/s12879-020-05164-7

**Published:** 2020-06-22

**Authors:** Mingliang Ying, Bin Lu, Jiangfeng Pan, Guanghong Lu, Shaobin Zhou, Dingjun Wang, Lu Li, Junkang Shen, Jiner Shu, From the COVID-19 Investigating and Research Team

**Affiliations:** 1 Department of Radiology, Second Affiliated Hospital of Soochow University, Suzhou, Jiangsu China; 2 Department of Radiology, Jinhua Municipal Central Hospital, Jinhua, Zhejiang China; 3 Ultrasound, Jinhua Municipal Central Hospital, Jinhua, Zhejiang China

**Keywords:** 2019-nCoV, COVID-19, Pneumonia, Computed tomography, Acute cholecystitis

## Abstract

**Background:**

The 2019 novel coronavirus (COVID-19) presents a major threat to public health and has rapidly spread worldwide since the outbreak in Wuhan, Hubei Province, China in 2019. To date, there have been few reports of the varying degrees of illness caused by the COVID-19.

**Case presentation:**

A case of 68-year-old female with COVID-19 pneumonia who had constant pain in the right upper quadrant of her abdomen during her hospitalization that was finally diagnosed as acute cholecystitis. Ultrasound-guided percutaneous transhepatic gallbladder drainage (PTGD) was performed, and the real-time fluorescence polymerase chain reaction (RT-PCR) COVID-19 nucleic acid assay of the bile was found to be negative. PTGD, antibacterial and anti-virus combined with interferon inhalation treatment were successful.

**Conclusion:**

The time course of chest CT findings is typical for COVID-19 pneumonia. PTGD is useful for acute cholecystitis in COVID-19 patients. Acute cholecystitis is likely to be caused by COVID-19 .

## Background

In December 2019, an outbreak of the 2019 novel coronavirus (COVID-19) occurred in Wuhan, Hubei Province, China, and rapidly spread throughout the world [1, 2]. Patients had clinical manifestations of fever, cough, and chest stuffiness in addition to other non-specific symptoms, including diarrhea, vomiting, abdominal pain and so on. Abdominal pain was uncommon [3]. Here, we report a confirmed case of a female with COVID-19 pneumonia who had constant pain in the right upper quadrant of her abdomen during her hospitalization that finally diagnosed as acute cholecystitis. Written informed consent was waived by the Jinhua Hospital of Zhejiang University Institutional Review Board.

## Case presentation

On January 31, 2020, a 68-year-old woman presented to the hospital with a 6-day history of fever, chest stuffiness and diarrhea without chills, cough, or nasal discharge. She had a history of good physical health and no underlying diseases. She had stayed with her son who had been diagnosed with 2019 novel coronavirus (COVID-19) pneumonia after business travel to Wuhan, China.

Her body temperature was elevated to 38.1 °C (100.6 °F) for 6 days before her hospitalization. The real-time fluorescence polymerase chain reaction (RT-PCR) assay of pharyngeal swabs and feces was positive for COVID-19 nucleic acid upon hospitalization. The patient’s temperature on admission was normal at 36.3 °C (97.3 °F), and there were coarse breath sounds from both lungs on auscultation. Laboratory studies showed a normal white blood cell count of 3.64 × 10^9^/L (normal range, 3.50–9.50 × 10^9^/L), differential neutrophil count of 70.7% (normal range, 40.0–75.0%), and lymphocyte count of 20.3% (normal range, 20.0–50.0%). The alanine aminotransferase (ALT), aspartate aminotransferase (AST), and total bilirubin levels were 19.0 (9.0–52.0) U/L, 27.0 (14.0–36.0) U/L, and 19.9 (3.0–22.0) μmol/L, respectively. There were elevated blood levels for C-reactive protein (33.7 mg/L; normal range, 0–10 mg/L).

**Fig. 1:**
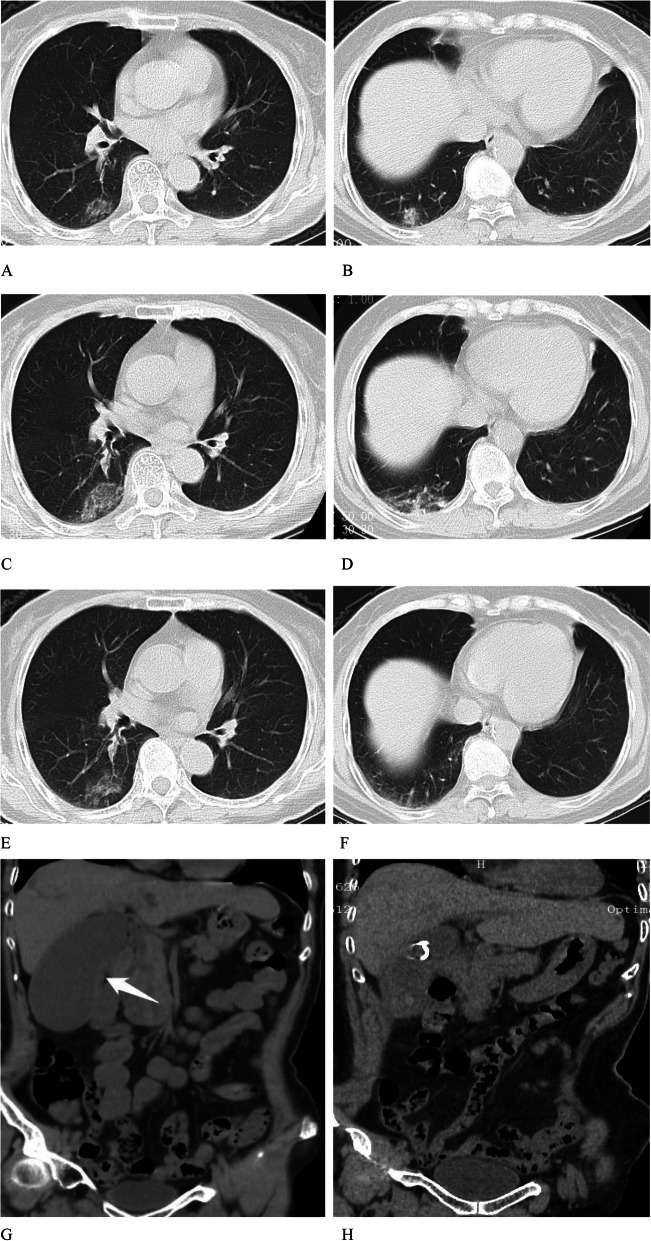
Chest and abdominal plain CT scans in a 68-year-old woman with COVID-19 pneumonia and acute csholecystitis. **a**, **b**. Transverse chest CT scan obtained on the first day after admission shows multifocal peripheral GGOs in the dorsal (**a**) and posterior basal (**b**) segments of the right lower lobe. **c**, **d**. Follow-up chest CT scan obtained on day 3 after admission shows progression of the GGOs. **e**, **f**. Follow-up chest CT scan obtained on day 13 after admission shows that the GGOs were partly resolved in the dorsal segment of the right lower lobe (**e**) and completely resolved in the posterior basal segment of the right lower lobe (**f**). **g**. Coronal MPR of the plain abdominal CT obtained on day 11 shows a distended gallbladder, hyperplasia of the gallbladder wall, biliary sludge (arrow), and no gallstones in the gallbladder. **h**. Coronal MPR of the plain abdominal CT obtained on day 13 shows shrinkage of the gallbladder or its change in shape, a high-density drainage tube and a little bleeding in the gallbladder. CT = computer tomography, GGOs = ground-glass opacities, MPR = multiplanar reconstruction

In the present case, the chest CT findings were typical for COVID-19 pneumonia (Fig. 1a-f). At first, the patient did not show any abdominal symptoms except diarrhea; however, she developed constant pain in the right upper quadrant of her abdomen and Murphy’s sign after 10 days of hospitalization, and her body temperature was elevated to 38.3 °C (100.9 °F). The laboratory examinations indicated elevated C-reactive protein (90.1 mg/L; normal range, 0–10 mg/L). We considered acute cholecystitis or cholangitis and performed an abdominal plain CT scan, which revealed a distended gallbladder, hyperplasia of the gallbladder wall and biliary sludge (Fig. 1g) and did not show gallstones in the gallbladder. Ultrasound-guided percutaneous transhepatic gallbladder drainage (PTGD) was performed on day 13, and approximately 500 ml of green gallbladder bile was drained on day 14. The RT-PCR COVID-19 nucleic acid assay of the bile was found to be negative. PTGD, antibacterial and anti-virus (lopinavir/ritonavir) combined with human interferon alfa-1b inhalation treatment were successful. The patient was subsequently discharged from the hospital on day 25 and referred to the clinic for follow-up.

## Discussion and conclusions

Since December 2019, a succession of patients in China have been suffering from pneumonia from unknown causes, later officially named COVID-19 by the World Health Organization, based on the whole genome sequence analysis of the viruses in respiratory samples or feces from the patients [4, 5]. Most early patients had a history of exposure to the South China Seafood Market in Wuhan city, and some patients demonstrated a family clustering feature. The clinical manifestations are mainly fever, fatigue, cough, and gradual dyspnea in some cases and acute respiratory distress syndrome in severe cases [3]. In the present case, the patient had a clear history of contact with the patient diagnosed with COVID-19 pneumonia, her lymphocytes were decreased, and the RT-PCR COVID-19 nucleic acid assay of pharyngeal swabs and feces was positive; thus, she was definitively diagnosed with COVID-19.

In the present case, the initial CT showed multifocal peripheral ground-glass opacities (GGOs) in the right lower lobe, which may represent hyaline membrane formation. Follow-up CT in this case demonstrated mild disease progression, as manifested by the increasing extent and multiple patchy consolidations, especially in the peripheral zones of the lungs, which may represent alveolar injury and inflammatory exudation. After symptomatic treatment, the consolidations and GGOs were almost absorbed, leaving cord-like shadows, which represent an improvement of the disease. The time course of lung changes on chest CT images of this patient were typical, as described in previous studies [6–8].

In our unique case, the patient developed constant pain in the right upper quadrant of the abdomen and Murphy’s sign after 10 days of hospitalization, and her body temperature was elevated to 38.3 °C. Because of the typical clinical presentation, this patient was likely to be diagnosed as acute cholecystitis or cholangitis in the clinic. Acute cholecystitis was subsequently confirmed by plain abdominal CT scan. It was difficult to identify the cause of acute cholecystitis in COVID-19 patients by abdominal CT imaging. And it could potentially cause severe gallbladder perforation, subsequently it was treated with PTGD. There has not been a study to report the RT-PCR nucleic acids for bile, while sputum or feces were with the highest positive rate of RT-PCR results. The precise mechanism of acute cholecystitis in COVID-19 patients was unknown. The RT-PCR results failed to find virus of COVID-19 in the bile. So our speculation about the potential association in the COVID-19 patient developed acute cholecystitis was a possible complication of COVID-19. However it remains to envisage acute cholecystitis as a possible complication of COVID-19, pending further studies that could prove or disprove this hypothesis.

It has not been reported whether the gallbladder might be vulnerable to COVID-19. We highlight a further complication possible with COVID-19. But the notable limitations of this study should be acknowledged. Only one unique COVID-19 patient presented an acute cholecystitis, the PCR test of the bile show no evidence of COVID-19 virus invasion of the gallbladder, and lack of pathological diagnosis for gallbladder tissue, which are not in favor of the possible association between COVID-19 and acute cholecystitis, it remains however possible that the infection by COVID-19 virus triggers the cholecystitis via a yet unknown mechanism.

In conclusion, we report the clinical course of a female patient with COVID-19. The time course of chest CT findings is typical for COVID-19 pneumonia. PTGD is useful for acute cholecystitis in COVID-19 patients. Acute cholecystitis is likely to be caused by COVID-19.

## Data Availability

The datasets used and/or analysed during the current study are available from the corresponding author on reasonable request.

## Abbreviations

ALT: Alanine aminotransferase
AST: Aspartate aminotransferase, aspartate aminotransferase
COVID-19: 2019 novel coronavirus
GGOs: Peripheral ground-glass opacities
PTGD: Percutaneous transhepatic gallbladder drainage
RT-PCR: Real-time fluorescence polymerase chain reaction
